# Oncolytic Adenovirus—A Nova for Gene-Targeted Oncolytic Viral Therapy in HCC

**DOI:** 10.3389/fonc.2019.01182

**Published:** 2019-11-08

**Authors:** Mubalake Abudoureyimu, Yongting Lai, Chuan Tian, Ting Wang, Rui Wang, Xiaoyuan Chu

**Affiliations:** ^1^Department of Medical Oncology, School of Medicine, Jinling Hospital, Nanjing University, Nanjing, China; ^2^Department of Medical Oncology, Jinling Hospital, Nanjing Clinical School of Southern Medical University, Nanjing, China; ^3^Department of Medical Oncology, Jinling Hospital, Nanjing, China

**Keywords:** gene-targeted oncolytic viral therapy, adenovirus, HCC, immunotherapy, virus engineering

## Abstract

Hepatocellular carcinoma (HCC) is one of the most frequent cancers worldwide, particularly in China. Despite the development of HCC treatment strategies, the survival rate remains unpleasant. Gene-targeted oncolytic viral therapy (GTOVT) is an emerging treatment modality—a kind of cancer-targeted therapy—which creates viral vectors armed with anti-cancer genes. The adenovirus is a promising agent for GAOVT due to its many advantages. In spite of the oncolytic adenovirus itself, the host immune response is the determining factor for the anti-cancer efficacy. In this review, we have summarized recent developments in oncolytic adenovirus engineering and the development of novel therapeutic genes utilized in HCC treatment. Furthermore, the diversified roles the immune response plays in oncolytic adenovirus therapy and recent attempts to modulate immune responses to enhance the anti-cancer efficacy of oncolytic adenovirus have been discussed.

## Introduction

Hepatocellular carcinoma (HCC) is one of the most frequent cancers worldwide and the third leading cause of cancer death. Even though great signs of progress have been made in standard therapies such as surgical removal, chemotherapy, and radiotherapy, the outcome remains disappointing. For this reason, the search for a new therapeutic approach is necessary. There is a bright new area in cancer-targeted therapy; “Gene Targeted Oncolytic Viral Therapy” (GTOVT) is considered a promising HCC therapy where oncolytic viruses are able to infect and kill cancer cells with no harm for normal cells (1). To date, a number of clinical trials have been conducted on oncolytic virotherapy for the treatment of HCC, as shown in Table 1. It is hoped that it will be used clinically in the future.

**Table 1:** Clinical trials using oncolytic adenovirus in HCC treatment.

	**ClinicalTrials.gov Identities**	**Status**	**Study title**	**Conditions**	**Interventions**	**Locations**	**Trial phase**	**Number of patients**	**Purpose**	**Genetic modifications**
1	NCT01869088	Unknown^†^	TACE Plus Recombinant Human Adenovirus for Hepatocellular Carcinoma	Hepatocellular Carcinoma	Drug: Recombinant Human Adenovirus Type 5 Injection Procedure: Transartery Chemoembolization	Cancer Center Sun Yat-sen University Guangzhou, Guangdong, China	Phase 3	266	Determine if TACE plus Recombinant Human Adenovirus Type 5 Injection will improve outcome in patients with advanced hepatocellular carcinoma (HCC) not amenable to surgery or local ablative therapy.	Ad5 E1B deleted
2	NCT03790059	Recruiting	Radiofrequency Ablation Combined With Recombinant Human Adenovirus Type 5 in the Treatment of Hepatocellular Carcinoma.	Hepatocellular Carcinoma	Drug: H101 Procedure: RFA	Institute of hepatobiliary surgery, Southwest Hospital Chongqing, Chongqing, China Institute of hepatobiliary surgery, Southwest Hospital Chongqing, China	Not Applicable	160	Retrospectively compare the short-term and long-term efficacy of RFA combined with H101 group and traditional RFA group in the treatment of small liver cancer (single lesion, diameter ≤ 3 cm).	H101
3	NCT03780049	Recruiting	HAIC Plus H101 vs. HAIC Alone for Unresectable HCC at BCLC A-B	Hepatocellular Carcinoma	Procedure: HAIC of FOLFOX Drug: H101 Drug: Placebos	Cancer Center Sun Yat-sen University Guangzhou, Guangdong, China	Phase 3	304	Evaluate the efficacy and safety of HAIC combined with H101 compared with HAIC alone in patients with unresectable hepatocellular carcinoma (HCC) at Barcelona clinic liver cancer A-B stage.	H101
4	NCT00844623	Completed	TK-based Suicide Gene Therapy for Hepatocellular Carcinoma	Carcinoma, Hepatocellular	Genetic: TK99UN	Clinica Universitaria de Navarra Pamplona, Spain	Phase 1	10	Determine whether activation of a prodrug after intratumoral gene transfer is safe in humans, and to determine dose levels for further clinical development.	Ad.TK encoding herpes simplex virus thymidine kinase
5	NCT02202564	Completed	Preliminary Results for the Double-dose Adenovirus-mediated Adjuvant Therapy Improving Outcome of Liver Transplantation in Patients With Advanced Hepatocellular Carcinoma	Liver Cancer Hepatocellular Carcinoma	Procedure: LT Drug: ADV-TK Drug: ganciclovir	Huazhong University of Science and Technology, Beijing Youan Hospital, China	Phase 2	81	Determine whether Adenovirus-mediated Adjuvant Therapy Improving Outcome of Liver Transplantation in Patients With Advanced hepatocellular carcinoma (tumors >5 cm in diameter,).	Adenovirus-thymidine kinase
6	NCT02561546	Unknown	p53 Gene Therapy in Treatment of Diabetes Concurrent With Hepatocellular Carcinoma	HCC Diabetes	Drug: p53 gene therapy Drug: Trans-catheter embolization	First affiliated hospital in Dalian University Dalian, Liaoning, China	Phase 2	40	Investigate preliminary efficacy using p53 gene therapy in treatment of diabetes concurrent with hepatocellular carcinoma (HCC).	rAd-p53
7	NCT00300521	Completed	Liver Transplantation With ADV-TK Gene Therapy Improves Survival in Patients With Advanced Hepatocellular Carcinoma	Hepatocellular Carcinoma Liver Transplantation	Genetic: ADV-TK (adenovirus-thymidine kinase enzyme) gene therapy	Beijing Chao Yang Hospital Beijing, Beijing, China	Phase 2	40	Determine whether ADV-TK gene therapy improving outcome of Liver Transplantation in patients with intermediate or advanced HCC.	ADV-TK (encoding adenovirus-thymidine kinase enzyme)
8	NCT03313596	Recruiting	Multicenter RCT of ADV-TK Gene Therapy Improving the Outcome of Liver Transplantation for Advanced HCC	Hepatocellular Carcinoma	Drug: ADV-Tk Procedure: LT	Beijing Youan Hospital Beijing, Beijing, China 301 Military Hospital Beijing, China General Hospital of Chinese People's Armed Police Beijing, China (and 8 more…)	Phase 3	180	Compare the effect of liver transplantation (LT) plus ADV-TK gene therapy vs. LT only in advanced primary hepatocellular carcinoma.	ADV-TK (encoding adenovirus-thymidine kinase enzyme)
9	NCT03563170	Recruiting	QUILT-3.072: NANT Hepatocellular Carcinoma (HCC) Vaccine	Hepatocellular Carcinoma Non-resectable Hepatocellular Carcinoma Recurrent	Biological: ETBX-011 Biological: GI-4000 Biological: haNK for infusion (and 15 more…)	Chan Soon-Shiong Institute for Medicine El Segundo, California, United States	Phase 1, Phase 2	382	Evaluate the safety and efficacy of metronomic combination therapy in subjects with advanced, unresectable, and untransplantable HCC.	
10	NCT02509169	Unknown^†^	Trans-catheter Arterial Embolization Combined With p53 Gene Therapy for Treatment of Advanced Hepatocellular Carcinoma	Advanced Hepatocellular Carcinoma (HCC)	Drug: TAE plus P53 gene Other: TAE	First affiliated hospital in Dalian University Dalian, Liaoning, China	Phase 1	60	Investigate clinical efficacy and immunoreaction using trans-catheter arterial embolization (TAE) combined with p53 gene therapy in treatment of advanced hepatocellular carcinoma (HCC).	rAd-p53
11	NCT02418988	Unknown^†^	Trans-catheter Chemo-embolization Combined With rAd-p53 Gene Injection in Treatment of Advanced Hepatocellular Carcinoma	Advanced Adult Hepatocellular Carcinoma	Drug: TACE plus rAd-p53 artery injection Drug: TACE	Xijing Hospital of the Fourth Military Medical University Xi An, Shanxi, China	Phase 1;	120	Investigate the efficacy and safety using TACE plus recombinant adenoviral human p53 gene (rAd-p53) in treatment of advanced HCC.	rAd-p5

† *Study has passed its completion date and status has not been verified in more than two years. Study was previously marked as Active, not recruiting*.

Oncolytic virotherapy was first reported a century ago, in 1912, by De Pace. He accidentally found that cervical cancer regressed in a patient who received Pasteur's attenuated rabies vaccine following a dog bite (2). In 2015, T-VEC (Imlygic^TM^), the oncolytic herpes virus, was approved in the western world for the treatment of advanced malignant melanoma (3) and became the first clinically used virus agent. In addition to the herpes virus, adenovirus, measles virus, reovirus, and Newcastle disease virus are used in oncolytic virotherapy. Among them, adenovirus vectors based on serotype 5 (Ad5) had been used widely due to several well-established advantages (4). First, a high titer of virus production can be easily produced [an average 10^12^ viral particles per milliliter (vp/ml) on each purification when 10^9^~10^10^(vp/ml) is used in clinic]. Second, they transduce both dividing and non-dividing cells efficiently. Most importantly, adenovirus backbones are flexible for genetic modifications. Thus, the adenovirus became the most feasible agent owing to its high degree of customizability (5, 6).

Oncolytic adenovirus anti-cancer efficacy is mostly determined by the host immune response. As for a pathogen, the adenovirus triggers a robust host immune response after infection. This kind of immune response is considered a “double-edged sword” as an oncolytic adenovirus stimulates both the anti-virus and anti-tumor immune response (7). On one hand, the host anti-virus immune response should be reduced to guarantee oncolytic virus cancer-killing efficacy. On the other hand, the anti-cancer immune response elicited by the oncolytic adenovirus expands its anti-cancer efficacy, including the metastasis side (8). In order to achieve the largest cancer-killing efficacy, it is necessary for GTOVT to construct an oncolytic adenovirus by genetic or non-genetic modification to reduce immunogenicity and to equip immunostimulatory molecules. In this review, we specifically summarized the recent progress of the oncolytic adenovirus utilized in HCC treatment, including direct oncolytic virotherapy and virus-based immunotherapy.

## Genetic Modifications of Oncolytic Adenovirus

**Figure 1:**
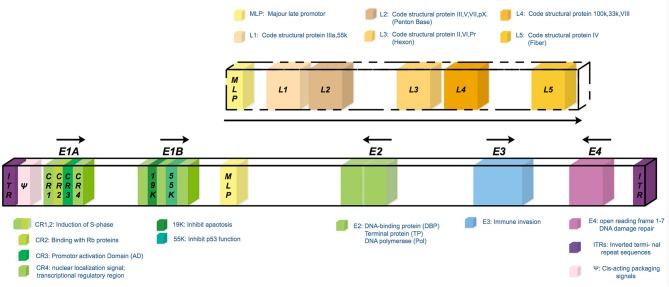
The scheme of the wild type serotype 5 adenovirus genome. The genome consists of four early transcription elements (E1, E2, E3, and E4) and five late expression genes (L1–L5) associated with adenoviral particle assembly. The E1 gene is required for the activation of the transcription of early genes; the E2 gene is required for virus DNA replication; the E3 gene is required for the modulation and evasion of the host's immune response, prevention of untimely cell death through apoptosis, and efficient cell lysis once new particle assembly is complete; the E4 gene is involved in virus RNA metabolism and transport, preferential downregulation of host-cell protein synthesis, and enhancement of virus DNA replication. The deletion of both the E1 and E3 genes can accommodate up to 7.5 kb of foreign DNA and is commonly used in gene therapy. E4 gene deletion further reduces induction of vector-specific immune responses and minimizes the outgrowth of replication-competent viruses in packaging cell lines.

Adenoviruses (Ads) are non-enveloped double-stranded DNA (dsDNA) viruses belonging to the Adenoviridae family (9). The Adenoviridae family is divided into seven species (A to G) (10) and includes 57 serotypes of the human virus. As for DNA viruses, the adenovirus has sufficient genome insertion capacity that is capable of carrying therapeutic genes of sizes of about 30–38 kb. Importantly, it could be expanded farther, up to 7.5 kb, by deleting viral genes such as the E3 gene (11). The Ads genome consists of four early genes (E1–E4), which are responsible for virus replication, and five late genes (L1–L5), code capsid proteins. The brief outline of each viral gene function is shown in figure below (Figure 1). At the onset of virus replication, the E1 gene is first expressed and then modulates other the expression of other virus genes. Thus, E1 gene deletion is commonly used in generating replication-deficient adenovirus vectors (11).

**Figure 2:**
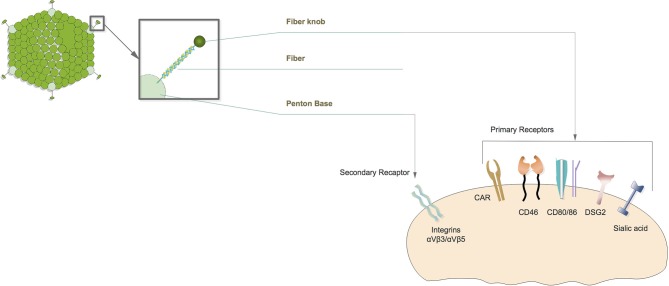
Diagram of oncolytic adenovirus infection. Oncolytic adenovirus infections initiated by fiber knob interact with the primary receptor on the cell membrane. Primary receptors are different according to adenovirus serotype. Afterwards, the penton base directly binds to secondary receptors that complete virus infection.

Adenovirus infection is dependent on cellular receptors that mediate the adenovirus's partial transfer into human cells (10) (Figure 2). In general, adenoviruses from specious A, C, E, and F use the coxsackievirus-Ad receptor (CAR), while serotypes from species B and D tend to use alternative receptors such as CD46, CD80, CD86, and DSG-2 (12). They all depend on Integrins as the same secondary receptor (13). Considering CAR is widely expressed in human epithelium cells, especially in the respiratory tract (14), wild type adenoviruses are not feasible agents for oncolytic virotherapy with the lack of tumor cell selectivity. Thus, a lot of efforts have been made to artificially modify adenoviruses for the purpose of gaining both tumor cell targeting and killing efficacy (15).

### Genetic Modifications to Achieve Cancer Selectivity

**Figure 3:**
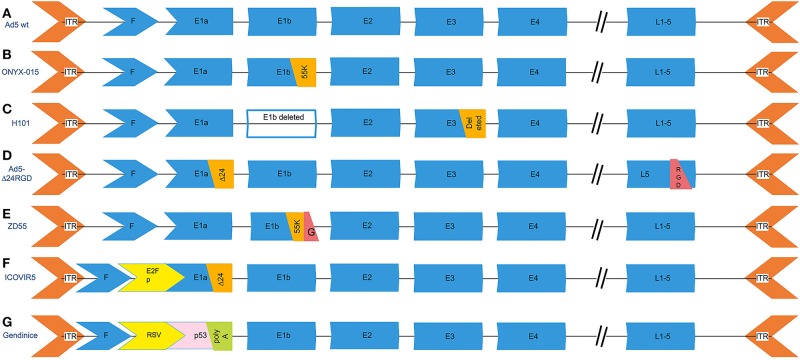
The genetic modifications of common oncolytic adenovirus vectors. **(A)** The genetic scheme of wild type serotype 5 adenovirus. **(B)** ONYX-015, the E1b-55k (2496-3323) region that is capable of replication in p53-deficient human tumor cells was deleted. **(C)** H101, deleting the entire E1B gene and a 78.3–85.8 nm gene segment in the E3 region, which is responsible for the code of the adenovirus death protein. **(D)** Ad5-Δ24RGD carries a 24-bp (919–943) deletion in the E1A region that is responsible for binding Rb protein and RGD motif insertion into the fiber. **(E)** ZD55 deleted the E1B 55-kD gene and armed with foreign gene. **(F)** ICOVIR5, the oncolytic adenovirus in which the endogenous E1A promoter has been replaced by the human E2F-1 promoter insulated with the DM-1. **(G)** Gendicine is a recombinant human serotype 5 adenovirus in which the E1 region is replaced by a human wild-type p53 expression cassette. Gendicine is a recombinant human serotype 5 adenovirus in which the E1 region is replaced by a human wild-type p53 expression cassette. The p53 gene is driven by a Rous sarcoma virus (RSV) promoter with a bovine growth hormone (BGH) poly(A) tail.

Cancer selectivity is an indispensable feature of oncolytic adenoviruses to ensure effective anti-cancer efficacy while avoiding unwanted side effects. Several studies have reported that tumor selectivity can be accomplished by deleting a part of viral genes (Figure 3). For example, the conversed region 2 (CR2) of the adenovirus E1A gene coding protein displaces RB and the RB-related proteins from the E2F transcription family in infected cells (16), which consequently derives quiescent cells entering S-phase (17). This process is essential for virus replication in normal cells. However, CR2 is dispensable in cancer cells due to them having sufficient free E2Fs (16). Zhang et al. had therefore constructed the AdCV101 with 24 bp deleted (922–946) in the CR2 region of the E1A gene. Their results showed that the AdCV101 exhibited less cytotoxicity in normal cells in comparison with HCC cell lines (17).

Furthermore, the E1B gene encodes two distinct tumor antigens, the 19-kDa (18) and 55-kDa proteins. The E1B-19 kDa protein is a homolog of Bcl-2 related apoptosis inhibitors (19–22). It has been demonstrated that the E1B-19KDa protein promotes cellular transformation by inhibiting E1A-induced apoptosis in a p53-independent manner (16). The E1B-55KDa protein is also capable of arresting the E1A apoptotic effect through p53-stabilization. Meanwhile, the E1B-55kDa protein forms a complex with the E4-orf6 gene product. Together, they mediate the viral mRNA accumulation and inhibit host cell mRNA transport and translation (23). leiOXYN-15, the E1B-55 kDa gene-deleted adenovirus, exhibited an excellent anti-cancer effect in clinical trials in head and neck cancer (24, 25). At first, its tumor selectivity was thought to be a consequence of inactivating mutations or deletions of the p53 gene in cancer cells (26, 27). However, OXYN-015 replication had been observed in several tumor cell lines that retain wild-type p53 sequences (28). These results made the precise role of p53 in ONYX- 015 selectivity more controversial. Later on, O'Shea et al. confirmed that the difference in RNA export between normal and tumor cells is a determinant of ONYX-015 oncolytic selectivity but not p53 inactivation (28). In addition, Sharon et al. explored that the E1b-55K domain is required for the inhibition of the host DNA damage response, which is important for adenovirus replication in HepG2 cells, and 2-Aminopurine can compensate for this domain loss (29).

However, the tumor selectivity of oncolytic adenoviruses that is produced simply by deleting viral genes is not qualified for avoiding unwanted tissue damage. Recently, cancer-specific promoters have become the most popular implement that controls oncolytic adenoviruses strictly targeted to cancer cells. In comparison to AdCN101, AdCN102, and AdCN103 exhibit an impressive reduction of virus replication in normal cells that were driven by an hTERT promoter (17). For liver cancer in particular, CV890, which was regulated by an AFP-promoter, selectively replicates in AFP-producing cells (30). The recently used oncolytic adenoviral vectors targeting HCC and their promoters are listed in the Table 2. In addition to the promoter itself, Kim et al. inserted a transcription enhancer to the distal region of a promoter to enhance transcriptional activity without specific loss (47). The apolipoprotein E (ApoE) promoter compensated for liver-specific PEPCK promoter transcription activity, which was hampered by a trans-splicing ribozyme (48).

**Table 2:** Recently studied oncolytic adenovirus vectors.

**Vector name**	**Type of gene**	**Armed gene**	**Function**	**Promotor**	**Ad backbone**	**Target**	**Reference**
Ad-sp-VGLL4	Tumor-suppressor	VGLL4	G2/M phase arrest; enhanced apoptosis	Survivin	Ad5	HCC	(31)
Ha2bm-d19	Transcription enhancer	a2bm; hypoxia-response elements (HRE)	Chimeric enhancer/silencer	AFP	Ad5	HCC	(32)
Ad.wnt-E1A (Δ24bp)-TSLC1	Tumor-suppressor	TSLC1 (tumor suppressor in lung cancer1)	Tumor suppressor gene which 5′ upstream region is methylated		E1A-Δ24bp	Targeting Wnt and Rb signaling pathway; cancer stem cell	(33)
SG655-mGMP		11R-P53; GM-CSF	11R: penetrating peptide	hTERT; MCMV	Ad5	Cancer stem cell;	(32)
No name	miRNA	miR-122	Negatively regulate virus replication	AFP	Ad5	AFP-positive; miR-122deregulated cell	(34)
QG511-HA- Melittin	Cytotoxic gene	Melittin	Water-soluble toxic peptide	Hybrid promoter, hypoxia-response element (HRE)-AFP promoter	ZD55	P53-deficient; AFP positive;	(35)
SG505-siFAK	Short-hairpin RNA	Hypoxia response element (HRE); siFAK	Against focal adhesion kinase (FAK)	hTERT; AFP promoter	SG505; Ad5	HCC	(36)
ZD55-XAF1	Increase sensitivity	XAF1 cDNA	Increase sensitivity		ZD55		(1)
GD55	Tumor targeting	GP73	Target HCC cells	GOLPH2 promote	ZD55	Liver cancer cells	(37)
AdCN305-SOCS1	Tumor-suppressor	SOC1 gene	Negative regulator of STAT pathway	hTERT; deletion of CR2 region		HCC	(38)
Ad-XAF-1&TNF-α	Tumor-suppressor	Ad-XAF-1&TNF-α	Inhibit proliferation	/	Ad5	HCC	(39)
AD55-Mn-SOD	Tumor-suppressor	Mn-SOD gene	Cell toxicity	AFP	ZD55	Liver cancer cells	(40)
SD55-TSLC1	Tumor-suppressor	TSLC1 (tumor suppressor in lung cancer1)	Tumor suppressor gene which 5′ upstream region is methylated	Survivin	ZD55	HCC	(41)
AFP-D55-SOCS3	Tumor-suppressor	SOCS3	Negative regulator of STAT pathway	AFP	ZD55	AFP-positive	(42)
SG600IL-24	Cytokine	IL-24	Immune response modulator	TERTp; HRE	ZD55	HCC	(43)
ADCN205	Cytokine	miRNA-34a & IL-24	Immune response modulator	Endogenous E3 promotor; hTERT for E1	Ad5-Δ24	HCC	(44)
Ad-ΔB/TRAIL	Cytokine	TRAIL	/	CMV promote E3	Ad-ΔB	HCC	(45)
Ad-ΔB/IL-12	Cytokine	IL-12	/	CMV promote E4	Ad-ΔB	HCC	(45)
SG511-CCL5-ODD	Chemokine	CCL5	Attracts immunocytes;	hTERT promote E1a, hypoxia-response element (HRE)-AFP promote E1b	Ad5	Tumor cell	(46)

In addition to active transduction selectivity, further ablation of the native tropism (for example, CAR-expressing normal cells) and/or modification of the vector to infect cells that are naturally not a target (for example, non-CAR-expressing cancer cells) is required. Fiber modification is commonly used to direct oncolytic adenovirus tumor tropism with the purpose of facilitating adenovirus transduction. The attachment of the globular knob domain on the adenovirus capsid protein to the cell surface CAR is the initial step of virus internalization (49). Thus, it is a basal element that mediates oncolytic adenovirus tumor-killing efficacy. Stepanenk et al. had classified recent modifications on the adenovirus vector fiber knob into five categories: (1) an incorporation of a targeting peptide into the fiber knob domain (the HI loop and/or C-terminus); (2) fiber knob serotype switching, or pseudotyping, by constructing chimeric fibers consisting of the knob domain derived from an alternate serotype (e.g., Ad5/3 or Ad5/35 chimeras [63#]), which binds to a receptor(s) other than CAR (e.g., desmoglein 2/DSG2 and/or CD46); (3) “fiber complex mosaicism,” an approach that combines serotype chimerism with peptide ligand(s) incorporation (e.g., Ad5/3-RGD); 4) “dual fiber mosaicism” by expressing two separate fibers with distinct receptor-binding capabilities on the same viral particle (e.g., Ad5-5/3 or Ad5-5/σ1); (5) fiber xenotyping by replacing the knob and shaft domains of wild-type Ad5 fiber protein with the fibrin itrimerization domain of a T4 bacteriophage or σ1 attachment protein of a reovirus (49). These modifications not only facilitate oncolytic adenovirus infection but may alleviate anti-virus immune responses that reduce virus immunogenicity by altering capsid antigens. However, it certainly had its limitation that the removal or exchange of virulence factors often limits virus replication (50).

### Genetic Modifications to Increase Cancer Cell Killing Efficacy

Other than cancer selectivity, oncolytic adenoviruses have one more indispensable feature, which is a strong cancer cell-killing efficacy. As described above, adenoviruses have enough genome capacity for a therapeutic gene vector. Furthermore, an increasing amount of research has reported that oncolytic adenoviruses are capable of infecting and expressing exogenous functional genes with no target cells. The functional/effector genes can be classified in four categories by their functions: (1) Tumor-suppressor gene (51, 52); (2) Cytotoxic gene; (3) Immune-modulating gene (53); (4) Tumor-antigens (54). The following is the detailed introduction of genes carried by oncolytic adenoviral vectors for HCC treatment.

#### Tumor Suppressor Genes

Vestigial-like (VGLL) proteins are transcriptional co-factors that have four members, VGLL 1–4. Among them, VGLL 4 has been studied exclusively. VGLL4 inhibits the YAP-TEAD transcription complex through direct competition with YAP for the binding of TEAD, which is the key member of the Hpo pathway (31). The widespread overexpression/overactivation of YAP has attracted much interest in VGLL4 as a therapeutic agent (55). It had been explored that VGLL4 has a tumor suppressor efficacy in lung, gastric, colorectal, and breast cancer (56–58). In addition to the transcription co-factor, Jin et al. found it is also a member of the binding Inhibitors of apoptosis proteins (IAP), which negatively regulate apoptosis (56). This evidence indicated that overexpression of VGLL4 in cancer cells might exhibit an anti-tumor effect, not only through YAP-dependent gene induction but also by the apoptosis pathway. Xie et al. had constructed an oncolytic virus Ad-sp-VGLL4 carrying the VGLL4 gene which regulated by survivin promotor that target to HCC cell line. As expected, Ad-sp-VGLL4 possesses a strong anti-tumor capacity. Their study first reported that over-expression of VGLL4 arrests the HCC cell cycle at the G2/M phase and restrains the S phase simultaneously. Therefore, there is a potential that Ad-sp-VGLL4 can be utilized for the treatment of HCC therapy (55). In addition to VGLL4, the other IAP-binding protein, X-linked inhibitor of apoptosis (XIAP)-associated factor 1 (XAF-1), is also regarded as a tumor suppressor. It had been discerned that X-linked IAP (XIAP) prevents the activities of caspase-3,−7, and−9 via directly binding to these caspases (59). The XAF-1 specifically inhibits XIAP and induces cancer cell apoptosis (60). Li et al. discovered that the co-delivery of XAF-1 and TNF-α via an adenovirus synergistically inhibits the apoptosis and proliferation of MHCC97L cells (39). They utilized 2A self-cleaved peptides to link XAF-1 and TNF-α coding sequences in one ORF, which is transcribed into one mRNA molecule, whereas it is translated into two different, function-independent proteins (61). Furthermore, it had been demonstrated that the constitutive expression of XIAP is also related to HCC resistance to Tumor necrosis factor-related apoptosis-inducing ligand (TRAIL) (42, 62, 63). Thus, Wei et al. utilized two independent oncolytic adenoviruses, AFP-D55-SOCS3 and AFP-D55-TRAIL (2:3 ratio), as a combined therapy for HCC. Their study showed that the restoration of SOCS-3 by an adenovirus antagonized TRAIL resistance by downregulating XIAP (64). SOCS-3 is a member of suppressors of cytokine signaling (SOCS) family, which consisting of eight members (CIS, SOCS-1 to 7) is well-investigated. The SOCSs, especially CIS, SOCS-1, SOCS-2, and SOCS-3, form a classical negative feedback circuit, inhibiting cytokine signaling pathways that are responsible for their induction. Particularly, SOCS-1 inhibits the JAK/STAT3 pathway by straightly binding to the JAK protein, while SOCS-3 binds to the activated receptor (38). As is well-known, JAK/STAT3 pathway activation is implicated in carcinogenesis. Therefore, AdCN305-SOCS1, the recombinant adenovirus expressing the SOSC-1 gene, exhibits an anticancer effect by inhibiting STAT3 phosphorylation and downregulating survivin, cyclin D1, Bcl-xL, and C-myc (65).

p53 is determined as a novel tumor-associated gene which mutations or deletions are related to most of the cancer formation. Since the frequency of p53 gene mutation/deletion is as high as 50.0% (average, 30.0%) in HCC (51), converting abnormal conformations of mutant p53 to normal p53 or enhancing the apoptosis of tumor cells by providing the exogenous p53 gene and the associated genes becomes a promising therapeutic strategy. The recombinant human wild-type p53-carrying adenovirus (Gendicine) is a pioneer gene therapy product approved for clinical use in China (66, 67). Aspp2 (apoptosis stimulating protein of p53-2) is one of the p53 binding proteins that enhances the p53 pro-apoptotic function (68). Interestingly, overexpression of Aspp2 presented anti-tumor effects in both a P53-dependent and independent manner (69). Aspp2 is therefore, to a certain extent, regarded as a tumor suppressor. Liu et al. demonstrated that Aspp2 increased colorectal cancer sensitivity to chemotherapy, especially to oxaliplatin, via inhibiting autophagy in a p53-independent pathway (70). Later on, they had generated a recombinant human adenovirus vector carrying the Aspp2 gene for use in HCC treatment in combination with oxaliplatin and found that Assp2-Ad inhibits hepatocarcinoma, independently of p53, in coordination with oxaliplatin (51).

#### Cytotoxic Genes

There are three types of primary intracellular antioxidant enzymes in mammalian cells: Superoxide dismutase (SOD), catalase, and peroxides. The SODs convert O-2 into H2O2, whereas the catalases and peroxidases convert H2O2 into water. The antioxidant enzymes are essential for preventing or repairing the damage caused by reactive oxygen species (71). It had been demonstrated that O-2 may act as a second messenger molecule to promote cell proliferation due to its generation markedly increased in ras-transfected fibroblasts (40). Huang et al. had found that the transduction of adenoviral MnSOD alone is enough to kill ras-transformed cells. They generated a dual-regulated AD55-Mn-SOD adenovirus, on the basis of a ZD55 backbone with E1B55-kDa-deletion of Ad5, and the E1a is regulated by a-fetoprotein (AFP) promoter and expresses superoxide dismutase (Mn-SOD). The AD55-Mn-SOD exhibited dramatical anti-cancer effects in HCC cells by activating the caspase apoptotic pathway (72). Furthermore, there were several studies reported that Mn-SOD gene therapy showed a novel anti-tumor effect also in pancreatic and colorectal cancer (71, 73).

However, the injection of AD55-MnSOD cannot achieve the complete inhibition of tumor cell growth, whether in tumor cells or at the animal level. Thus, it is necessary to explore the combination of this with other therapeutic strategies, for example, dual-gene-viral therapy, conventional chemotherapy, or immunotherapy (72).

## Modulation of the Host Immune Response Triggered by Oncolytic Adenovirus Therapeutics

Despite its increasing advantages, gene-targeted oncolytic virus therapy is not popularized. This is because the current study found that gene monotherapy based on an oncolytic adenovirus is insufficient for tumor eradication. That the oncolytic adenovirus limited anti-tumor efficacy is attributed to several reasons. First, the majority of the human population had anti-adenovirus immunity, which significantly hampers the oncolytic adenovirus efficacy by causing rapid virus clearance after systemic administration (74). Second, the virus-mediated inflammatory response may cause severe complications that impairs safety (75). Third, the viral antigens might be presented by target cells after virus transfection and may ultimately induce a cytotoxic cellular response (76). In short, the adenovirus-triggered host immune response is one of the determining factors for its anti-cancer efficacy.

As far as virus-mediated immune responses are concerned, they include both host anti-virus and anti-tumor immune responses. The virus-based immunotherapy is the delivery of virus vectors harboring immune stimulatory factors to alleviate anti-virus immune responses as well as boost anti-tumor immunity in the tumor milieu.

### Anti-virus Immune Response

Viral vectors could trigger a host anti-virus immune response, which intensely limits their applicability in several ways. First, viral vectors stimulate the expression of neutralizing antibodies (NAbs) against structural components due to their intrinsic immunogenicity (77, 78). The Pre-existing NAbs dramatically inhibit adenoviral vectors efficacy, and this leads to therapy termination. Second, intravenously administered adenoviruses are rapidly cleared by the mononuclear phagocytic system (MPS) in the liver and spleen (4).

To tackle this problem, multiple strategies, such as switching Ad serotype, fiber-nob modification, and constructing a hybrid vector system combining viral and non-viral carriers, are obliged to avoid virus-mediated immune responses Among them, serotype switching is the least reliable because each type of Ads has multiple surface-exposed capsid proteins containing neutralizing epitopes (79). An increasing amount of studies are committed to constructing a hybrid vector system with non-genetic modifications. The aim of the hybrid vector system is to construct adenoviruses “invisible” to immunogenic effector cells and components to thereby prolong blood circulation time and reduce immunogenicity (80), for instance, Polymeric adenoviruses engineered chemically or physically with numerous polymers coating on the virus surface. Commonly used chemically conjugated polymers, like poly(ethylene) glycol (PEG) (81) and the arginine-grafted bioreducible polymer (ABP) (82) have higher serum stability, while physically engineered polymers have better transduction efficiency. Oncolytic adenoviruses coated by polymers through electronic interaction are easy to manipulate since the cationic polymers with anionic adenovirus can form Ad/polymer complexes without a chemical reaction. Furthermore, it had been demonstrated that net cationic surface-charged Ads increase virus cellular uptake and transgene expression (83). However, this character causes non-specific viral uptake that might result in an unwanted side effect (80). Chen et al. engineered the oncolytic adenovirus PLC-ZD55-IL-24 and were intended to combine the advantages of both. The cationic calcium phosphate, which had been demonstrated to induce adenovirus cellular uptake and viral gene expression (84), was coated on an ionic virus capsid by electronic interaction. Then, to remedy the limitation of cationic polymers that lack specificity, the virus surface is stabilized by a neutrally charged PEG via Dioleoylphosphatydicacid (DOPA)er. Not only the negligible immune response was observed following the systemic administration of PLC-ZD55-IL-24 but it showed an efficient tumor-targeting ability and anti-tumor effect against HCC (85).

### Anti-tumor Immune Response

Interestingly, immunosuppressed patients generally respond better to oncolytic virus therapy than those with an intact immune system and that this higher oncolytic activity is often associated with unacceptable toxicity. However, suppression of the adaptive immune response is a double-edged sword. To our knowledge, tumor immune evasion is the main obstacle restricting anti-tumor therapies. Tumor cells escape from immune responses in various ways, including through the secretion of immune-suppressing cytokines, the deficient expression of immune-modulating cytokines, and the recruitment of immune-inhibitory cells. Oncolytic adenoviruses provide a new strategy that combine tumor-debulking activity and the anti-tumor immune response. This combination therapy not only facilitates virus anti-tumor activity in the primary cancer milieu, but it also targets the metastatic lesion by activating the anti-tumor immune response. In order to modulate adaptive anti-tumor immunity, immune stimulatory, and/or co-stimulatory cytokines expressing Ads have been investigated in HCC immunotherapy.

IL-24 is one of the most potent immune-stimulatory cytokines and is a member of the IL-10 class-II cytokine family. In addition to modulating the immune response, IL-24 also showed anti-proliferative effects and induced cell apoptosis even with the genetic defects including the alteration of p53, Rb, and p16/INK4a (86, 87). Its anti-proliferative effect had been found in variety of cancer types, such as ovarian cancer (88, 89), lung carcinoma (90, 91), breast cancer (92), pancreatic cancer (93), glioma (94), prostate cancer (95), and colon cancer (96). SG600-IL24 is IL-24 expressing an oncolytic adenovirus generated based on the ZD55 backbone with a telomerase reverse transcriptase promoter (TERTp) that controls the E1gene and Hypoxia regulatory elements (HRE) that control the E1a and E1b gene (43). Xue et al. had studied the ability and function of SG600-IL24 to express IL-24 in HCC cell lines. Their study showed that SG600-IL24 can selectively suppress the proliferation and induce apoptosis of HCC cell lines *in vitro* but not normal liver cell line L02 in a p53-independent manner (97).

Liu et al. had developed the Cancer-targeting dual gene-viro-therapy (CTGVT-DG) strategy, which combines two tumor-suppressor genes that may have a compensative or synergetic effect (98). They investigated the combined therapy with IL-24 and the tumor necrosis factor-related apoptosis-inducing ligand (TRAIL), which was considered as a promising anti-tumor agent expressed in two Ads, respectively (99). Their study showed that a combination of two anti-tumor genes (IL-24 with TRAIL) may be a promising strategy for gene-viro therapy, which exhibits a synergistic anti-tumor effect (98). Furthermore, the combination therapy of Ad-ΔB/IL-12 with Ad-ΔB/TRAIL exhibits an enhanced anti-tumor immune response due to IL-12 being able to upregulate the TRAIL expression of NK cells, resulting in IFN-γ-dependent NK cell-related tumor metastasis inhibition (45, 100). Co-therapy with IL-12 and TRAIN complements TRAIL mono therapy poor pharmacokinetic property and induce HCC cells sensitivity to TRAIL's apoptotic effect (101). In addition, the enhanced anti-tumor efficacy of SG600-IL24 was observed in combination with IFN-α (102).

However, it had been found that some tumor cells over-expressed anti-apoptotic protein Bcl-2 and antagonized the IL-24 function (44). Thus, to recover IL-24 pro-apoptotic efficacy, Lou et al. constructed an AdCN205-IL-24-miR-34a that expressed both IL-24 and miRNA-34a (103). Previous studies demonstrated that miRNA-34a directly regulates the Bcl-2 (104). Significant induced tumor suppression and reduced expression of Bcl-2 had been observed after AdCN205-IL-24-miR-34a infection in comparison with AdCN205-IL-24 or AdCN205-miR-34a alone *in vitro* and vivo. In conclusion, downregulation of Bcl-2 induced by miRNA-34a can overcome tumor cell resistance to IL-24 and enhanced its anti-tumor effect (103).

In addition to IL-24 and IL-12, Sun et al. investigated whether recombinant adenoviruses expressing IL-2 (rAd-IL-2) as a gene immunotherapy agent could optimize the prognosis of HCC patients (53). IL-2 treatment was the first immunotherapy approved by the US Food and Drug Administration for use in melanomas (105). Recently, it had been demonstrated that oncolytic adenoviruses express interleukin-2 (IL-2), and the tumor necrosis factor alpha (TNF-a) can achieve an anti-tumor immunomodulatory effect similar to lymphodepletion. Importantly, using an oncolytic adenovirus is much safer than Lymphodepleting preconditioning with high-dose chemotherapy (106). According to Sun et al.'s study, rAd-IL-2 exhibits a significant induced anti-tumor immune response by recruiting CD4^+^ and CD8^+^T cells, increasing the interferon-γ release, and stimulating cytotoxic T lymphocyte responses in the HCC tumor model (53).

## Oncolytic Adenovirus in Pre-Clinical Studies

Thanks to the advantages of oncolytic adenoviruses, a number of pre-clinical studies have been conducted on HCC treatment. As early as 2006, it was reported that improved recurrence-free survival and the overall survival had shown in advanced HCC patients receiving adjuvant ADV-TK (adenovirus vector expressing herpes simplex virus thymidine kinase) Gene Therapy after liver transplantation, as opposed to those who received liver transplantations alone (107). The feasibility and safety of intra-tumoral administration of an adenoviral vector encoding for HSV-TK were assessed in phase 1 clinical trials in HCC patients (108). Recently, the preliminary results from the phase 2 clinic trial declared that the double-dose adenovirus-mediated adjuvant therapy improved the outcome of liver transplantation in patients with advanced HCC (109). The other ongoing clinical trials are detailed in Table 1. Besides the anti-tumor properties of the oncolytic adenovirus itself, its combination with other agents has been studied and found to increase the cancer-killing efficacy. For example, the synergistic efficacy of a chemodrug, such as 5-FU, Gemcitabine, doxorubicin, and Paclitaxel (PTX), used in combination with an oncolytic adenovirus has been recorded (110). A phase 3 clinic trial of Hepatic artery infusion chemotherapy (HAIC) of FOLFOX in combination with oncolytic adenovirus in HCC treatment is under recruitment (NCT03780049). Furthermore, the compatibility of oncolytic adenoviruses with immune stimulator factors, like IL-2,-12, and−24, and immune checkpoint inhibitors, like (PD-1)/CD137L (111), has been studied recently. The combination of the anti-PD-1 antibody (Pembrolizumab) and Ad5-Δ24-RGD (DNX-2401) is being evaluated in a Phase II clinical trial for the treatment of glioblastoma (NCT02798406). There is no data for the use in HCC at present.

## Discussion

Gene-targeted oncolytic virus therapy (GTOVT) had innovated a brand-new area in cancer treatment that provides the possibility of targeting all cancer types by designing viral vectors. The adenovirus is the most promising agent used in GTOVT due to several good characteristics. Theoretically, gene-armed viral vectors could target all type of cancers, including both solid tumors and hematological malignant ones. In spite of clinical trials having confirmed the safety of viral agents in clinical use, oncolytic adenoviruses have not yet been used in clinic. This is due oncolytic adenoviruses being strictly inhibited during systemic delivery *in vivo*, which hampers their effectiveness. To overcome this shortcoming, bioengineering, molecular, and immunological approaches have been considered. For example, decorated oncolytic adenovirus vectors are produced by genetic &/or non-genetic (capsid modification by polymers, like PEG, etc.) tools to avoid neutralization by anti-virus antigens and serum components and to prevent sequestration by MPS in the liver and spleen, which is the other major reason for virus clearance from circulation. Furthermore, high interstitial pressure and a tight stromal extracellular matrix (ECM) blocks the virus spread into the tumor foci. These problems are partially solved by the oncolytic adenovirus being armed with functional genes, such as Hyaluronidase or Metalloproteinase, or delivered by MSCs as a virus carrier for inducing virus uptake. It should be noted that some transgene expression may result in the loss of viral fitness. All these stations result in premature the clearance of the oncolytic adenovirus through a substantial immune response (112). Therefore, the oncolytic Ad-based immunotherapeutics have attracted increased interests recently due to their ability to modulate the anti-tumor immune response and evade the anti-virus immune response. However, finding the balance between the anti-tumor immune response and anti-virus immune response is a key issue that remains unsolved.

## Author Contributions

MA: manuscript drafting and figure design. TW and RW: manuscript editing and revision. XC: literature review. YL and CT: clinical trials review and table design. RW: research team management and oversight.

### Conflict of Interest

The authors declare that the research was conducted in the absence of any commercial or financial relationships that could be construed as a potential conflict of interest.
