# Corrigendum: Oncolytic Adenovirus—A Nova for Gene-Targeted Oncolytic Viral Therapy in HCC

**DOI:** 10.3389/fonc.2020.00701

**Published:** 2020-05-05

**Authors:** Mubalake Abudoureyimu, Yongting Lai, Chuan Tian, Ting Wang, Rui Wang, Xiaoyuan Chu

**Affiliations:** ^1^Department of Medical Oncology, School of Medicine, Jinling Hospital, Nanjing University, Nanjing, China; ^2^Department of Medical Oncology, Jinling Hospital, Nanjing Clinical School of Southern Medical University, Nanjing, China; ^3^Department of Medical Oncology, Jinling Hospital, Nanjing, China

**Keywords:** gene-targeted oncolytic viral therapy, adenovirus, HCC, immunotherapy, virus engineering

In the original article, we neglected to include the funder the Excellent Youth Foundation of Jiangsu Province, China, BK20140032 to Rui Wang.

The authors apologize for this error and state that this does not change the scientific conclusions of the article in any way. The original article has been updated.

